# Reviews

**Published:** 1887-07

**Authors:** 


### REVIEWS.

THE ARCHIVES OF VETERINARY SCIENCE, published by the Medical Department of the Ministry of the Interior St. Petersburg, Russia.

The February number of the above mentioned review, now before us, constitutes the first issue in the present year of the bi-monthly publication of extensive veterinary records, being conducted under the auspices of the St. Petersburg Ministry of the Interior for the last seventeen years, the subscription price for the publication being five roubles for the Government Veterinary Surgeons and students, and seven roubles for the ordinary public.

This publication is the official organ of the Russian Ministry of the Interior, rendered necessary for the promulgation of Government rules concerning veterinary supervision to be enforced throughout the empire, the transportation of cattle within the limits of the country, the killing off and disposal of the bodies of animals succumbing to epizootic diseases, as well as for the publication of the last veterinary police measures adopted at the last deliberation of the Veterinary Surgeons in the principal cities of the empire and approved by the Ministry of the Interior. Those measures are always communicated officially by the Ministry of the Interior to all Governors of the Provinces—or, as they are called "governments" of Russia— who have to enforce their adoption in their parts of the country, while the insertion of the test of the same measures in the "Veterinary Archives" brings the same to the notice of all people interested in the subject throughout the Empire. The "Veterinary Archives" present also the lists of most recent promotions among the Veterinary Surgeons of Russia, as well as the reports concerning the government Institutes of Veterinary Science in the two capitals and the largest cities of the Empire, such as Kharkov, Kazan, Dorpat, Warsaw, Odessa, etc.

PART I. The official part of the magazine is given up entirely to official communications concerning the latest Veterinary police measures, the spreading or localization of epizootic diseases, changes in the Veterinary Department of the Ministry, etc.

PART II. *Biological: Professor E. K. Brandt* on the "Animal parasites of domestic mammalians and birds." (Illustrated.)

PART III. *Pathologo-therapeutic and surgical. Professor G. Poluta*, paper on "Physiological effect of laxative mineral drugs."

*Professor V. E. Vorontzov* on the report of Prof. Williams and Prof. Roberts in the British National Veterinary Society.

*Popoff, V. S.* on "Inflammatory prolobi in ducks."

*A. Gryshin, V. S.* on "Epidemic abortions among cows, attributable to their feeding on moulded oat-straw."

PART IV. *Hygenic and Rural Economic. P. M. Medvedsky,* "Horse breeding in Russia."

*K. T. Kalning,* "The growth of the hoof and its acceleration by irritating applications."

*A. T. Jacobson and M. A. Ignatief,* "A project of measures for the prevention of contamination with the Siberian plague of men working on animal products in factories, etc."

PART V. *Veterinary Police and Judiciary. Prof. E. M. Zemmer,* "Symptomatic carbuncle in horses." (Illustrated.)

*A. N. Trussof,* "Law of June 3d, 1879, in the government of Kaluga. Deliberations on the introduction of that law in the Southern governments."

PART VI. *Bibliographical.* Protocols of the sittings of the Society of Veterinary Surgeons in St. Petersburg.

*V. G. Tatarsky,* "The activity of the S. V. S. in Moscow in 1885 and 1886. Scientific reports of the Veterinary Institute of Kazan."

PART VII. *Miscellaneous.* Containing reviews of the foreign medical periodicals and books.

A TREATISE ON DIPHTHERIA. Historically and Practically Considered, including Croup, Tracheotomy and Intubation. By A. Same, Docteur en Medicine, etc., etc. Paris. Translated, Annotated and the Surgical Anatomy added. Illustrated with a full page colored lithograph, and many wood engravings. By Henry Z. Gill, A. M., M. D., LL. D. Cleveland, Ohio. Price (cloth) $5.00. St. Louis, Mo., J. H. Chambers & Co., 1887.

The above is the title of an extremely valuable, sensible, and interesting work on that much dreaded disease diphtheria, which has just issued from the press of Messrs. J. H. Chambers & Co., of St. Louis, Mo. As the able translator, Dr. Gill has so aptly said: "The book grows in interest, until it becomes a charm." Dr. Same has treated his subject in a masterly and exhaustive manner, beginning far back in its earliest history, and tracing it down to the present day. He discusses in a clear and able fashion, the many theories that have attached themselves to diphtheria, and as would be expected, he disposes of most of them as useless, empirical, and not proven. In this we regard him as eminently sound. He clears away the rubbish of his subject, and presents the digested facts, in a plain, convincing and simple manner. We heartily agree with him when he says that diphtheria, "Is a disease unique, and specific," that it is contagious, and is a *constitutional* disease, the pharyngeal diphtheric membrane, being only a local expression of a general malady. He lays proper stress upon local treatment selecting such remedies only as experience has proved to be useful, and dismisses with a few sensible words, the great host of drugs that have been recommended by the inexperienced, and that encumber our literature, Constitutional treatment justly holds a high place in his estimation. Appreciating the severe depression that comes on so early in diphtheria, he advocates supporting measures, and gives to alcohol the prominence it really deserves. His ideas on these points are very clear, and very ably and forcibly set forth. The subject of Tracheotomy occupies a considerable space in this work, and here again, Dr. Same stands upon sensible and rational, as well as scientific ground. Before this desperate and dangerous expedient is resorted to, he asks us to consider well, all the grave factors in the problem, and while it is an established surgical procedure, and sometimes imperatively needed, yet in the majority of cases, it is of doubtful utility, and its sequelæ are so numerous and severe, that we are rarely justified in its performance. The work is rich in statistics and records of cases, and is a valuable contribution to the literature of this terrible disease.

The chapter on the Surgical Anatomy of the Pre-Tracheal Region, with special reference to Tracheotomy in children is of great value as clearly illustrating the anomilies of the vessels and nerves of that important anatomical region. The materials for this chapter have been freely taken from the works of Dr. L. S. Pilcher of Brooklyn, N. Y. The publishers have spared no pains in making the book an attractive volume, and the medical profession may congratulate itself that so clear and able a work has been added to its literature. H. W. M.

THE PROCEEDINGS OF THE LINNEAN SOCIETY OF NEW SOUTH WALES. (Second Series.) Vol. I. Part IV. for the year 1886. Sidney, 1887.

This volume of nearly 300 pages, contains the papers, twenty-three in number, read in the last three months of 1886, and the address of the President, Prof. W. J. Stephens, M. A., F. G. S., at the Annual General Meeting. It is a good indication of the interest taken by our Antarctic friends in the Sciences, and especially in Natural History.

In the *Mammalia* a new species of *Hapalotis* from N. W. Australia is described.

In *Reptilia* a new fresh water Tortoise, *Carettochelys inscueptus* and two new species of Geckos, *Nephurus levis* and *Diplodactylus toniconda* are described.

The President in his address dwelt on the importance of making the study of Natural History as an essential part of general education. He deplored the fact that Science was not pursued for the sake of Science in the educational establishments of the country at large. He says, he is convinced that such a pursuit is natural and lawful and even, to some extent, obligatory upon civilized men, and that it fails to be recognized in that light entirely on account of a pedantic, irrational and unnatural system of public education. Compared with Physical Science, how much easier its study. No artificial apparatus is necessary ; we are always in the Laboratory. The most tremendous yet, to us the most trivial phenomena are incessantly appealing to our senses and our intellect, and it is only our schooling that renders us apathetic, blind and deaf, and dead to their challenge,

W. A. C.

Oxygen in Therapeutics, by C. E. Ehinger, M. D. Cloth, 12 mo., pp. 156. Chicago, W. A. Chatterton & Co., 1887.

Just now, there is a good deal of therapeutic oxygen in the air. Perhaps there is no single element which has been so variously, and for the most part, quackishly advertised. Literally, millions of bottles of some sort of solution, which probably contained about the usual proportion of combined oxygen—*aqua impura*, for instance—have been sold at a verv exorbitant price during the past ten years, and the leading journals and magazines, daily, weekly and monthly, exalt the virtues of oxygen, until intelligent people have become fairly disgusted. The very word oxygen, has, to many physicians, come to be almost synonymous with quackery. Nevertheless, oxygen has a field as legitimate as that of any other agent or element.

Dr. Ehinger has not attempted an original work, and does not claim to have been a pioneer in this field. He is rather a compiler of the more recent labors of others who have been too timid or too busy to place themselves on record in any more permanent form than fugitive papers in the medical journals. The book is a modest and practical work, and will be an aid to those who would learn what has been done by a legitimate use of oxygen and allied gases, used as remedial agents.

It would be strange, if such a work should be free from errors. There are some crude chemical statements, some evidently typographical blunders, but it is suggestive of the possibilities of the oxygen group, when it shall be systematically studied by those, whose sole object is not the harvest of dollars that jingle in the pockets of successful charlatans. It will throw some light on a subject hitherto, and still not well understood.

